# Expanding Ayurvedic Pharmacology: Ecological, Quality Standards, and Environmental Health Implications of Medicinal Plant Drug Discovery

**DOI:** 10.7759/cureus.110701

**Published:** 2026-06-11

**Authors:** Mayuri Dani, Meenu Srivastava Khare, K. Parameswaran Namboothiri, Shravani Girish Kulkarni, Saniya Yusufkhan Pathan, Vaishnavi G Kulkarni, Aashish Patel

**Affiliations:** 1 Department of Rasashastra and Bhaishajya Kalpana (Iatrochemistry and Pharmaceutical Sciences), Maharashtra University of Health Sciences, Nashik, IND; 2 Department of Rachana Sharir (Anatomy), Shri Narayan Prasad Awasthi Government Ayurved College, Raipur, IND; 3 Department of Panchakarma (Ayurvedic Detoxification and Rejuvenation Therapy), Amrita School of Ayurveda, Amrita Vishwa Vidyapeetham, Amritapuri, IND; 4 Department of Rachana Sharir (Anatomy), Maharashtra University of Health Sciences, Ayurved Seva Sangh's Ayurved Mahavidyalaya, Nashik, IND; 5 Department of Balrog (Paediatrics, Ayurveda), College of Ayurved, Bharati Vidyapeeth (Deemed to be University), Pune, IND; 6 Department of Rasayana and Vajikaran (Rejuvenative and Reproductive Medicine), KAHER's Shri BM Kankanawadi Ayurveda Mahavidyalaya, Belgaum, IND; 7 Department of Kayachikitsa (Internal Medicine), KAHER's Shri BM Kankanawadi Ayurveda Mahavidyalaya, Belgaum, IND

**Keywords:** agro-ecology, ayurveda, biodiversity conservation, environmental health, medicinal plants, sustainable sourcing

## Abstract

The growing global use of Ayurveda has increased demand for medicinal plants used in preventive, therapeutic, and integrative healthcare. This expansion has strengthened Ayurvedic pharmacology but has also raised ecological, quality-control, and environmental health concerns, particularly where botanicals are obtained from wild or weakly monitored supply systems.

This review examines sustainable medicinal plant sourcing as a central requirement for biodiversity conservation, raw material authenticity, and public health protection in Ayurvedic drug discovery. It focuses on therapeutic expansion, disease-specific pharmacological applications, phytochemical evidence, conservation pressure, agro-ecological cultivation, authentication, contamination control, and safety assessment.

The review concludes that environmental sustainability should be treated as a determinant of medicinal plant quality because overharvesting, habitat degradation, adulteration, species substitution, and contamination can compromise both ecological stability and therapeutic reliability. Sustainable cultivation, botanical repositories, traceability systems, toxicity screening, efficacy-oriented quality grading, and community-based resource governance are recommended to support safe, authentic, and therapeutically consistent Ayurvedic herbal medicines.

## Introduction and background

Plants have long been central to preventive, promotive, and curative medicine in Ayurveda, where they serve as primary constituents of many formulations [1]. This historical role remains important because Ayurvedic formulations continue to depend on botanically derived raw materials. Current pharmaceutical literature shows that Ayurveda-related medicinal plants are now being investigated for hypertension, asthma, skin diseases, diabetic wound healing, cancers, Alzheimer’s disease, and infectious diseases [2]. This expansion has increased research interest in both Ayurvedic pharmacology and plant-derived bioactive agents [3]. The growing role of medicinal plants in integrative medicine has also encouraged evaluation of traditional medicines through pharmacognosy, phytochemistry, molecular docking, network pharmacology, and clinical approaches [4]. Ayurvedic pharmacology is increasingly relevant to multi-target drug discovery because many medicinal plants contain diverse secondary metabolites [5]. Antihypertensive plants are being studied for their possible effects on metabolism, oxidative stress, inflammation, and vascular function [2]. Plant-based medicines for asthma may act through anti-inflammatory, bronchodilatory, immunomodulatory, and antioxidant mechanisms [6]. Ethnodermatological studies show the continued contribution of Ayurvedic preparations and medicinal plants to dermatological healthcare in India [1]. Medicinal plants have also been reviewed for diabetic wound healing because they may support antimicrobial activity, collagen synthesis, angiogenesis, and anti-inflammatory responses [7].

The significance of Ayurvedic medicinal plants has evolved further in dermatology and cosmetology, cancer, neurodegeneration, and antiviral studies. In cosmetology, Ayurvedic preparations and botanicals may be translated through standardised herbal formulations for skin protection, wound repair, pigmentation control, anti-inflammatory care, antioxidant support, and ageing-related skin concerns [1]. This translation requires pharmacognostic authentication, phytochemical profiling, safety testing, formulation standardisation, and clinical evaluation so that traditional topical and oral preparations can be developed into evidence-based dermatological and cosmeceutical products [4]. Studies reviewing Ayurvedic anticancer drugs highlight the involvement of plant-based medicines in apoptosis induction, modulation of oxidative stress, cell cycle regulation, and immune response pathways [8]. Recent studies have evaluated the complementary role of Ayurveda in prostate cancer therapy by focusing on symptom relief and quality of life measures [9]. Computational studies have also revealed the involvement of Ayurvedic medicinal plants as dual-target drug candidates for Alzheimer’s disease, suggesting their potential use in neurodegenerative diseases [10]. The COVID-19 pandemic has increased the significance of Ayurvedic medicinal plants since multiple studies have analysed the role of these plants for antiviral activities [11]. Molecular docking studies have assessed plant-based medicines such as *Withania somnifera*, *Tinospora cordifolia*, and *Ocimum sanctum* against SARS-CoV-2 main protease [12].

Despite progress in pharmacological research, the ecological and sourcing dimensions of Ayurvedic pharmacology have received comparatively less attention. This gap is important because the therapeutic value of Ayurvedic botanicals depends not only on pharmacological activity but also on how raw materials are identified, harvested, processed, and supplied. Herbal drug safety and reliability depend first on accurate species identification and the correct selection of the plant part used [13]. Harvest time, geographical origin, and processing methods further influence the quality of the raw material [13]. Phytochemical composition is also important because variation in active constituents can affect therapeutic consistency. The pharmacological properties of *Bacopa monnieri* illustrate this relationship, as its clinical relevance depends on extraction quality, phytoconstituent profile, safety, and bioavailability [13].

The rising pharmaceutical importance of medicinal herbs can increase pressure on plant populations when commercial demand exceeds natural regeneration capacity [14]. Such pressure links Ayurvedic drug discovery directly with biodiversity conservation, environmental exposure, and public health risk. Overexploitation, habitat destruction, and informal trade can contribute to biodiversity loss and possible contamination of medicinal plants [5]. Adulteration or misidentification of raw materials may reduce therapeutic efficacy because Ayurvedic drugs depend on precise botanical identity and consistency. Contaminated soil, polluted irrigation water, chemical pesticide use, and poor post-harvest handling may introduce hazardous substances into herbal remedies [7]. Therefore, sustainable procurement should integrate ecological conservation with raw material authentication, contamination control, traceability, and community-based governance [3]. This review specifically examines how medicinal plant sourcing practices affect the quality, safety, therapeutic reliability, and environmental health accountability of expanding Ayurvedic pharmacology.

Objective of the review

The objective of this narrative review is to examine how medicinal plant sourcing affects the ecological sustainability, raw material quality, safety, and environmental health accountability of expanding Ayurvedic pharmacology. The review specifically focuses on biodiversity conservation, resource pressure, authentication, contamination risks, quality control, and sustainable procurement practices for Ayurvedic herbal medicines.

## Review

Methodology

This narrative review was developed through a structured literature search focused on Ayurvedic pharmacology, medicinal plant sourcing, phytochemistry, quality control, authentication, conservation, contamination risk, and environmental health. PubMed/MEDLINE, Scopus, Web of Science, ScienceDirect, and Google Scholar were searched for literature published between 2003 and 2026, with priority given to recent and directly relevant studies. Search terms included combinations of “Ayurveda,” “Ayurvedic pharmacology,” “medicinal plants,” “phytochemistry,” “pharmacognosy,” “network pharmacology,” “molecular docking,” “drug discovery,” “sustainable sourcing,” “biodiversity conservation,” “agroecology,” “botanical authentication,” “quality control,” “adulteration,” “contamination,” “toxicity,” and “environmental health.”

Studies were included if they addressed pharmacological applications of Ayurvedic or Indian medicinal plants, phytochemical evidence, disease-specific therapeutic relevance, sustainable cultivation, conservation, authentication, traceability, toxicity, contamination, or environmental health implications. Reviews, original research articles, computational studies, ethnopharmacological studies, and conservation-focused publications were considered. Studies were excluded if they lacked relevance to medicinal plant pharmacology, sourcing, quality, safety, or sustainability. Evidence was selected and appraised narratively according to relevance, methodological clarity, recency, and contribution to the review objective. No meta-analysis or formal risk-of-bias assessment was performed.

Review

Ayurvedic Pharmacology and Therapeutic Expansion

Ayurvedic pharmacology is increasingly being examined through modern biomedical approaches because medicinal plants contain multiple phytoconstituents that may act on interconnected molecular targets [4]. This feature is consistent with classical Ayurvedic practice, where polyherbal formulations are commonly used, and therapeutic effects are often understood as the result of combined actions rather than isolated single compounds [14]. Ayurvedic preparations may contain several active ingredients, and these compounds can produce primary therapeutic effects as well as secondary biological effects on related pathways. This complexity makes Ayurvedic therapy a multidisciplinary field that requires pharmacology, phytochemistry, toxicology, formulation science, clinical evaluation, and quality assurance to be considered together. Network pharmacology has therefore become useful for analysing AYUSH-recommended immune-supportive plants by mapping interactions among plant compounds, disease pathways, and immune-related targets [4]. These findings suggest that Ayurvedic plants may exert activity through synergistic interactions among several phytoconstituents rather than through a single active compound [3]. The evidence supporting this therapeutic expansion, however, is not uniform. Computational screening, molecular docking, and network pharmacology can identify plausible plant-compound-target relationships, but these methods mainly provide hypothesis-generating evidence [15]. Molecular docking and dynamics studies of active molecules from Ayurvedic herbal drugs against SARS-CoV-2 targets indicate growing interest in computational approaches within Ayurvedic pharmacology [11,15]. Docking studies involving *W. somnifera*, *T. cordifolia*, and *O. sanctum* also show how traditional medicinal herbs are being explored in antiviral drug-discovery campaigns [10,12]. These findings should be interpreted as mechanistic or preclinical evidence rather than proof of clinical efficacy. Computational work cannot substitute for controlled clinical trials, but it can help prioritise compounds, guide experimental validation, and clarify possible mechanisms for further pharmacological studies [12].

Ayurvedic pharmacology is also evolving through advances in classification, authentication, and quality assurance. The chemosensory classification of medicinal plants using an electronic tongue and multivariate analysis shows that traditional sensory parameters can be converted into measurable analytical fingerprints [16]. This is important because Ayurvedic drug identification has historically relied on sensory and experiential assessment, whereas contemporary quality assurance requires reproducible analytical confirmation [6]. Several approaches can help bridge this gap between traditional pharmacology and current quality-assurance standards. These include pharmacognostic authentication, macroscopic and microscopic identification, phytochemical fingerprinting, chromatographic profiling, DNA barcoding, metabolomics, chemosensory analysis, contamination testing, toxicity screening, batch-to-batch standardisation, and traceable sourcing documentation [16]. Such approaches do not replace traditional knowledge; rather, they provide verifiable tools to confirm botanical identity, composition, safety, and consistency. Disease-focused formulation research further illustrates the need to distinguish between levels of evidence. Reviews on medicinal plants with antihypertensive effects describe pharmacognostic, phytochemical, and pharmacological characteristics that may support formulation development through a multidisciplinary approach [17]. Similarly, Ayurvedic plants have been discussed as potential anticancer agents through mechanisms such as apoptosis, oxidative stress modulation, cell signalling, and immunomodulation [8,9]. These mechanistic observations are valuable, but they should not be interpreted as equivalent to clinical efficacy in cancer, hypertension, respiratory disease, diabetes, neurodegeneration, or infectious disease. The translational pathway should therefore proceed from traditional use and phytochemical evidence to preclinical testing, safety evaluation, formulation standardisation, and well-designed clinical studies.

**Figure 1:**
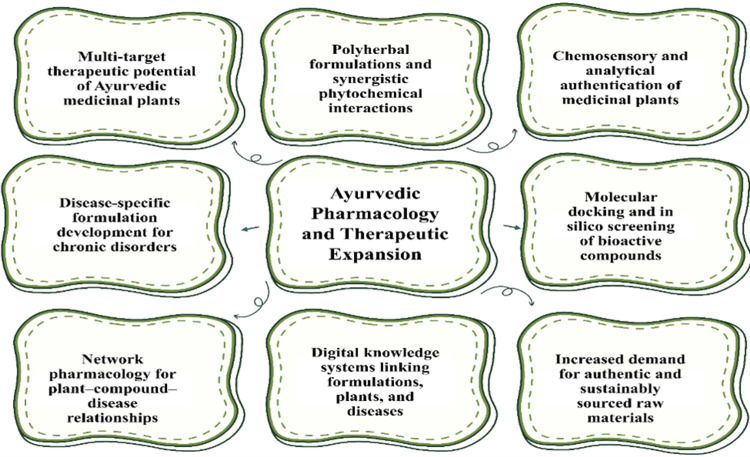
Conceptual framework of Ayurvedic pharmacology and therapeutic expansion This image is an original author-created schematic using Microsoft PowerPoint (Microsoft® Corp., Redmond, WA, USA) and was not generated using AI.

Digital knowledge systems may strengthen this translational pathway by improving the organisation and verification of Ayurvedic pharmacological information. GRAYU integrates formulations, medicinal plants, phytochemicals, and disease information into a graph database that supports systematic investigation of traditional medicine knowledge [18]. These databases can improve research transparency by structuring associations among plant species, formulations, bioactive compounds, and disease indications [18]. Overall, the current literature suggests that Ayurvedic pharmacology is moving toward an integrative model that combines traditional knowledge, phytochemistry, bioinformatics, analytical quality assurance, and pharmacological validation [3]. While pharmacological investigations continue to expand the therapeutic scope of Ayurvedic medicinal plants, their translational value depends on factors beyond biological activity alone. Clinical applicability requires reliable species identification, phytochemical consistency, contaminant control, and sustainable access to raw materials. Consequently, advances in molecular pharmacology, computational screening, and disease-specific applications should be interpreted alongside considerations of medicinal plant conservation, sourcing practices, quality assurance, and environmental health. Figure 1 presents a conceptual framework linking multi-target activity, polyherbal phytochemical interactions, chemosensory authentication, molecular docking, network pharmacology, disease-specific formulation development, digital knowledge systems, and demand for sustainable raw materials. This framework supports the central argument that the expansion of Ayurvedic pharmacology should be evaluated not only by therapeutic potential but also by authentication, reproducibility, sourcing reliability, and environmental accountability.

Disease-Specific Pharmacological Applications

Disease-specific research on Ayurvedic and medicinal plants has expanded across chronic, inflammatory, metabolic, neurologic, respiratory, and cancer-related conditions [3]. In hypertension, medicinal plants are mainly discussed in relation to vascular reactivity, oxidative stress, endothelial function, inflammation, and metabolic regulation [2]. These mechanisms are relevant because hypertension involves multiple interacting pathways rather than a single pathological target [19]. Reviews on antihypertensive medicinal plants therefore support their role as potential sources for cardiovascular drug development, although clinical translation requires standardised formulations and stronger efficacy evidence [17]. In respiratory disease, medicinal plants are commonly evaluated for asthma-related mechanisms such as bronchodilation, mast cell stabilisation, anti-inflammatory activity, antioxidant defence, and immune modulation [5,6]. These mechanisms correspond to the major pathological features of asthma, including airway inflammation, airway hypersensitivity, mucus secretion, and immune dysregulation [5]. The repeated appearance of anti-inflammatory and antioxidant activity across disease areas suggests that many botanicals may act through shared biological pathways, but these effects should still be interpreted according to disease-specific models and evidence strength [3].

Medicinal plants have also been evaluated for diabetic wound healing and other metabolic complications [13]. In diabetic wounds, delayed repair is commonly associated with infection, oxidative injury, vascular insufficiency, impaired collagen formation, and chronic inflammation [7,13]. Reviews suggest that medicinal plants may support healing through antimicrobial, antioxidant, anti-inflammatory, pro-angiogenic, and collagen-promoting effects [7]. One relevant example is berberine, an isoquinoline alkaloid reported from plants such as *Berberis aristata*, *Berberis vulgaris*, *Coptis chinensis*, and *T. cordifolia*. Berberine has been studied for diabetic wound repair because of its antimicrobial, anti-inflammatory, antioxidant, and tissue-repair-related effects [7,15]. Medicinal plants and plant-derived compounds may therefore be useful in wound healing models when evaluated under appropriate safety, dosing, formulation quality, and clinical-validation standards [20]. Neurological disorders represent another area of expanding interest [21]. Natural medicines have been investigated for Alzheimer’s disease because plant-derived compounds may influence oxidative stress, cholinergic neurotransmission, protein aggregation, neuroinflammation, and neuroprotection [15,21]. Research in this area has also examined molecular targets and the difficulty of converting bioactive plant chemicals into effective drugs [22]. Recent computational analysis has identified Ayurvedic medicinal plants as possible sources of dual-targeting compounds for Alzheimer’s disease [10]. These findings are useful for hypothesis generation, but they should not be interpreted as equivalent to clinical efficacy without experimental validation and controlled human studies. Such results clearly indicate that, besides being traditional medicines, medicinal plants may become interesting pharmaceutical targets based on disease mechanisms [23]. Ayurvedic and medicinal plants show disease-specific pharmacological relevance across several conditions (Table 1).

**Table 1:** Disease-specific pharmacological applications, evidence level, and translational considerations of Ayurvedic and medicinal plants

Disease / Clinical Area	Pharmacological Relevance	Major Mechanistic Focus	Evidence Type / Strength	Key Limitations and Translational Concerns	Reference
Hypertension	Medicinal plants reviewed as potential antihypertensive agents.	Vascular tone, oxidative stress, inflammation, and metabolic regulation.	Mainly review-based pharmacognostic, phytochemical, and pharmacological evidence.	Requires standardised formulations, dose validation, safety assessment, and stronger clinical evidence before therapeutic translation.	[2]
Asthma	Medicinal plants have been studied for asthma-related therapeutic effects.	Bronchodilation, anti-inflammatory activity, antioxidant action, and immune modulation.	Primarily traditional-use, review-based, and preclinical/mechanistic evidence.	Findings should not be interpreted as clinical efficacy without controlled studies; sourcing consistency is needed to ensure reproducible phytochemical profiles.	[6]
Diabetic wound healing	Medicinal plants examined for delayed wound repair in diabetes.	Antimicrobial activity, collagen formation, angiogenesis, and inflammation control.	Mainly review-based and experimental wound-healing evidence.	Clinical translation requires wound-specific safety testing, formulation standardisation, contamination control, and validated dosing.	[7]
Cancer	Ayurvedic plants reviewed as potential anticancer agents.	Apoptosis, oxidative stress modulation, cell-cycle regulation, and immune response.	Mostly mechanistic, preclinical, and review-based evidence.	Anticancer relevance should be interpreted cautiously because mechanistic activity is not equivalent to clinical efficacy; toxicity and herb-drug interaction risks require evaluation.	[8]
Alzheimer’s disease	Ayurvedic medicinal plants explored for dual-target anti-Alzheimer compounds.	Target-based computational screening for neurodegenerative drug discovery.	Predominantly in silico drug-discovery evidence.	Computational findings are hypothesis-generating and require experimental validation, bioavailability assessment, toxicity testing, and clinical studies.	[10]
COVID-19	Traditional Indian medicinal plants reviewed for possible COVID-19 management.	Antiviral potential and immune-supportive effects.	Mainly a systematic review of traditional, preclinical, and supportive evidence.	Evidence should be distinguished from proven antiviral efficacy; authentication, quality control, and safety monitoring are essential for public-health use.	[11]

Cancer-based uses have likewise gained prominence in the literature surrounding Ayurveda and medicinal plants [8]. There have been publications regarding the use of Ayurveda in the treatment of prostate cancer, specifically in an integrative approach dealing with symptom relief, support treatments, and quality-of-life concerns [9]. Reviews about Ayurvedic plants as potential anticancer drugs focus on how they might be involved in processes such as apoptosis, oxidative stress, cell cycle control, and immune response mechanisms [8]. Reviews concerning lung carcinoma include information about medicinal plants as well as green-synthesised treatments for cancerous conditions [14]. Disease-specific reviews like these show the scope of treatment by medicinal plants but emphasise the importance of authentic and sustainable raw materials [3].

Key Ayurvedic Medicinal Plants and Phytochemical Evidence

Many of the Ayurvedic medicinal plants are currently part of modern pharmacological research, as their traditional uses are getting supported by phytochemical, preclinical, clinical, and computational evidence [13]. A prominent Ayurvedic medicinal plant to be studied for neuroactivity and cognition is *B. monnieri* [3,13]. Its therapeutic value is generally associated with bacosides, antioxidant properties, neuroprotection, and potential cognitive enhancement [13]. Recent reviews also stress the need for extract standardisation, bioavailability, dose, safety, and maintenance of consistent phytochemical quality to determine the clinical usefulness of *B. monnieri* [24]. Another important Ayurvedic plant with wide-reaching value is *W. somnifera* (Ashwagandha) [25]. The rational use of this plant has been reviewed in terms of health support, adaptation to various pressures, rejuvenation, inflammation, and restorative medicines [25]. There have also been reviews on the potential use of *W. somnifera* in neurodegenerative diseases, in particular, its role in modulating oxidative stress, protecting cells from injury, and disease-modifying properties [26]. It represents a classical Ayurvedic medicine that is now a subject of pharmacological research, but requires systematic consideration of aspects such as species ascertainment, cultivation, extract composition, and safety [4,26].

Another herb that has attracted much pharmacological interest is *O. sanctum* (Tulsi) for its antioxidative, anti-inflammatory, immunomodulatory, and antiproliferative effects. Commentaries about its use as an "elixir of life" show that it remains significant in traditional and modern therapies [27]. Tulsi's phytochemical variability provides evidence for its investigation in multiple biomedical applications, though its broad therapeutic potential requires standardisation of preparation and uniform starting material [27,28]. Equally, liquorice, in Ayurveda called "Yashtimadhu", has been assessed using metabolomics and network analysis, demonstrating how plant omics can be used to understand plant phytochemistry and activity [28]. Other plants show the diversity of phytochemical data for Ayurvedic and ethnomedicines. The metabolic diversity and potential of *Holarrhena pubescens* have been reviewed to highlight its role as an ethnomedicinal plant containing several bioactive compounds [29]. Biochemical studies of the methanolic extract of *Evolvulus alsinoides* have also attracted interest for their phytochemistry and bioactivity [30]. Aquilaria trees producing agarwood have also been reviewed for natural products, chemistry, biological activities, and biosynthesis, revealing the broader importance of medicinal and aromatic plants in drug development [31]. Important medicinal plants have phytochemical and medicinal significance in modern pharmacological research (Table 2).

**Table 2:** Ayurvedic medicinal plants, active constituents, evidence level, and translational relevance

Medicinal Plant / Ayurvedic Name	Active Constituent / Alkaloid and Source	Major Evidence Focus	Pharmacological Relevance	Key Translational or Sustainability Concern	Reference
Bacopa monnieri	Bacosides from *Bacopa monnieri* whole plant/herb	Pharmacological attributes and clinical manifestation.	Cognitive and neuroprotective relevance.	Clinical translation depends on extract standardisation, bioavailability, dose consistency, and safety evaluation.	[13]
*Withania somnifera* / Ashwagandha	Withanolides from roots and leaves of *Withania somnifera*	Rational Ayurvedic use for health and healing.	Stress adaptation, vitality, restoration, and health promotion.	Requires standardised preparation, toxicity assessment, and consistency between root- and leaf-derived products.	[25]
*Ocimum sanctum* / Tulsi	Eugenol, ursolic acid, and rosmarinic acid from leaves of *Ocimum sanctum*	Therapeutic anticancer potential.	Antioxidant, anti-inflammatory, immunomodulatory, and anticancer relevance.	Mechanistic anticancer findings require cautious interpretation and stronger preclinical and clinical validation.	[27]
Liquorice / Yashtimadhu	Glycyrrhizin and liquiritin from the roots of *Glycyrrhiza* species	Metabolomics and network pharmacology.	Phytochemical diversity and biological activity.	Quality control should address species identity, metabolomic variability, dose-related safety, and herb-drug interaction risk.	[28]
*Tinospora cordifolia* / Giloy	Berberine, palmatine, and magnoflorine alkaloids from stems of *Tinospora cordifolia*	Phytochemistry, ethnopharmacology, clinical application, and conservation.	Immune-supportive and broad Ayurvedic pharmacological relevance.	High demand requires authentication, conservation-sensitive sourcing, and careful safety monitoring.	[12]
Saraca asoca	Flavonoids, tannins, catechin, and epicatechin from the bark of *Saraca asoca*	Traditional uses, biological activities, and conservation.	Important conservation-sensitive Ayurvedic medicinal plant.	Bark harvesting creates sustainability concerns; cultivation and substitution control are needed for a reliable supply.	[32]

These examples show that Ayurvedic medicinal plants are biologically complex resources whose therapeutic value depends not only on reported pharmacological activity but also on species identity, genotype, active constituent profile, plant part used, growing environment, harvesting stage, extraction method, formulation context, safety assessment, and strength of evidence [28]. Phytochemical evidence strengthens the scientific basis of Ayurvedic pharmacology, but it also highlights the need for authentication, standardisation, and sustainable sourcing [29]. If medicinal plant demand increases without ecological safeguards, the quality and availability of these resources may decline [31]. Plants such as *T. cordifolia* and *Saraca asoca* are especially relevant because increasing therapeutic and commercial demand may intensify pressure on natural populations. Therefore, phytochemical research should be integrated with conservation planning, cultivation protocols, traceable supply chains, quality-oriented raw material management, and conservation-sensitive procurement [24].

Sustainable Cultivation and Agro-Ecological Sourcing

Sustainable cultivation is essential for conserving medicinal plant resources while supporting the continued development of Ayurvedic pharmacology [16,32]. A transdisciplinary agro-ecological approach can integrate biodiversity conservation, farmer livelihoods, ecological resilience, and medicinal plant quality [32]. Agro-ecological sourcing is important because phytochemical quality depends on growing conditions, harvesting practices, and post-harvest processing [33]. This approach can reduce pressure on wild populations while improving the reliability of raw materials used in Ayurvedic formulations [34]. It is necessary to emphasise cultivation-based sourcing where high commercial and therapeutic demands exist, such as for *T. cordifolia* [35]. Reviews of *T. cordifolia* provide information about its phytochemistry, ethnopharmacology, clinical use, and conservation practices [35]. It becomes evident that there is a need to match the therapeutic popularity of the plant with its proper sustainable use [4]. This will ensure the sustainability of the plant in nature while meeting commercial demand.

Sustainable sourcing is also necessary for critically endangered plant species, such as *S. asoca* [36]. Extensive studies have been conducted on the traditional uses, biological activity, historical significance, and conservation relevance of *S. asoca* [3]. Given that the use of medicinal plants involves the utilisation of the bark of these and other species, there is a risk of pressure on natural populations if harvesting is not properly managed. Cultivation, along with conservation-based harvesting practices, will mitigate this issue [32].

**Figure 2:**
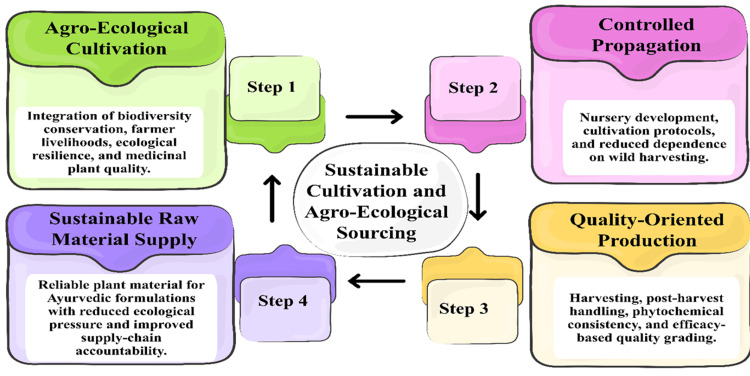
Key components of sustainable cultivation and agro-ecological sourcing of medicinal plants This image is an original author-created schematic using Microsoft PowerPoint (Microsoft® Corp., Redmond, WA, USA) and was not generated using AI.

Efficacy-driven cultivation is another essential element of sustainable sourcing [33]. The development of an efficacy-based quality grading system, which was developed based on the case study of *Phyllanthus emblica*, demonstrated the importance of linking the quality of medicinal plants with specific measures of efficacy and chemistry [9,33]. Grading systems will facilitate informed decisions during the procurement of plant materials, as they will help distinguish raw materials based on their quality in terms of efficacy [33]. In turn, this concept is particularly pertinent to Ayurvedic pharmacology [16]. Botanical repositories also support conservation by preserving verified reference material for medicinal plant species [34]. The creation of a harmonised repository for medicinal plants in India would help to conserve the samples for reference purposes, identity certification, and even facilitate conservation-related research [34]. This is because there are many middlemen involved in medicinal plant supply chains, making it difficult to identify the exact species involved or to detect substitution of plant species [18]. Pharmacological literature has pointed out that research in the field of medicinal plants should take into account ecological and quality issues alongside increasing demand [3]. Sustainable sourcing should include cultivation, conservation, quality grading, botanical authentication, and community participation [32]. Examples such as *T. cordifolia*, *S. asoca*, and *P. emblica* demonstrate that therapeutic demand can increase pressure on medicinal plant supply systems unless cultivation and conservation strategies are developed together [33,35,36]. Agro-ecological cultivation can reduce dependence on wild harvesting and improve raw material consistency [32]. Environmental sustainability should therefore be regarded as a determinant of medicinal plant quality [34]. Sustainable cultivation and agro-ecological sourcing comprise several interlinked activities (Figure 2).

Conservation, Biodiversity, and Resource Pressure

Conservation is central to the future of Ayurvedic pharmacology because medicinal plant products depend on viable ecosystems, stable plant populations, and sustainable harvesting systems [32]. Globally, more than 1,300 medicinal plants are used in Europe, and about 90% of these are harvested from wild resources; in addition, up to 80% of people in developing countries depend on herbal medicines for primary healthcare, and more than 25% of prescribed medicines in developed countries are derived from wild plant species [35]. These figures show that medicinal plant conservation is not a marginal ecological issue but a major requirement for healthcare supply, pharmaceutical development, and public health security. Expansion in herbal medicine use may increase pressure on species that are difficult to replace, especially those with narrow habitats, slow growth, or long maturation periods [36]. Plants harvested for bark, roots, rhizomes, resin, or other non-renewable parts are particularly vulnerable because regeneration can be slow after collection [36]. Biodiversity loss may therefore affect raw material availability, species authenticity, phytochemical consistency, and long-term supply reliability [34]. Biodiversity banks of Indian medicinal plants can support authentication, quality assurance, harmonisation, and conservation by linking plant identity with verified reference material [34]. When species are substituted, misidentified, or collected without ecological documentation, both conservation and medicine safety are weakened [34].

Species-specific reviews indicate that several Ayurvedic medicinal plants require conservation efforts [35]. The conservation aspects of *T. cordifolia* have been evaluated together with phytochemistry and clinical applications, suggesting that popularity among therapeutic preparations may lead to resource exploitation [7,35]. Similarly, *S. asoca* has been studied from an ethnopharmacological perspective with respect to its use, biological activity, and conservation [36]. It is especially significant for sustainable cultivation and harvesting due to the risk of depletion of natural populations and substitution of raw materials in the marketplace [36]. There are instances of medicinal and aromatic plants that are also under habitat pressure [31]. Although chemistry, biological effects, and biosynthesis of compounds from agarwood and *Aquilaria* species have been reviewed, these plants face additional challenges arising from the high economic value of their products in the marketplace [32]. Such situations pose the problem of increasing pressure on raw material extraction unless conservation practices are adopted [33].

In addition, several Ayurvedic plants require careful consideration of conservation issues, since their demand could increase due to newly acquired pharmacological importance [37]. In fact, *Lepadaneria reticulata* is one such species, as it has already undergone evaluation in regard to its phytochemistry and pharmacological importance [36]. Such scientific interest might lead to a better understanding of the therapeutic use of these plants; however, it could also increase the demand for their raw materials [37]. Frameworks for quality grades of plants, such as for *P. emblica*, may help relate their quality to sustainability [33]. The issue of conservation cannot be divorced from cultivation strategy either. Cultivation of medicinal plants from an agroecological perspective ensures not only biodiversity, farmers' participation, ecological resilience, and stable raw material production [32]. Botanical libraries, quality grades, and conservation-based cultivation methods may serve as a means to enhance the base of medicinal plant resources [33]. Consequently, sustainable procurement must provide for the conservation of wild plants as well as the development of cultivation methods to sustain their use by pharmaceutical companies [38].

Quality Control, Authentication, and Safety

Quality control is central to Ayurvedic pharmacology because the safety and efficacy of medicinal plants depend on accurate botanical identification, phytochemical consistency, contamination prevention, and post-market quality monitoring [34]. Herbal product reliability may be compromised by substitution, misidentification, contamination, improper processing, dose variability, or exposure to polluted environments [4]. Botanical repositories for Indian medicinal plants can support authentication by providing taxonomic verification, pharmacognostic assessment, conservation documentation, and quality assurance [34]. Such resources can reduce confusion related to plant identity and improve confidence in Ayurveda-based products. However, authentication alone does not ensure clinical safety. Even correctly identified botanicals may pose risks if they contain heavy metals, pesticide residues, microbial contaminants, variable concentrations of active constituents, or compounds capable of herb-drug interactions.

The problem of authentication can be addressed by incorporating instrumental methods into traditional approaches for identifying medicinal plants [6,32]. Electronic tongue analysis with multivariate statistics has shown that sensory categorisation of plants can be converted into measurable analytical fingerprints [16]. Analytical fingerprinting can also support batch-to-batch consistency and facilitate pharmacological investigation and clinical translation [16]. Systems such as GRAYU, a graph-based knowledge resource integrating Ayurvedic formulations, medicinal plants, phytochemicals, and diseases, may assist researchers and pharmaceutical producers in verifying relationships among species, phytochemicals, and medical applications [18]. This is important in a field where traditional names, regional substitutes, and formulation variations can create identification problems.

Clinical safety evaluation must extend beyond botanical authentication and preliminary bioactivity testing. Safety evaluation studies involving in vitro screening of medicinal plants against uropathogens have also assessed cytotoxicity using brine shrimp lethality assays [20]. Such tests provide useful preliminary information on biological activity and toxicity potential, but they cannot establish clinical safety by themselves [16]. A more complete safety framework should include heavy metal testing, pesticide residue analysis, microbial limit testing, aflatoxin screening, dose standardisation, herb-drug interaction assessment, adverse event reporting, and post-market surveillance. These measures are necessary because medicinal plants can have strong biological effects, and sustainable sourcing or correct identification does not automatically guarantee therapeutic reliability or patient safety [20].

Standardisation is crucial even in widely used herbs such as *B. monnieri*, because therapeutic reliability depends on consistent extracts, phytochemical profiles, safety, and bioavailability [24]. If extracts are not standardised, variation in active constituents can create inconsistencies in pharmacological and clinical studies [24]. Efficacy-based grading frameworks, such as those proposed for *P. emblica*, show that herbal quality should be assessed by therapeutic relevance rather than only by appearance or commercial grade [33]. Quality standards should also address environmental safety because medicinal plants may accumulate contaminants from soil, irrigation water, pesticides, storage, or processing methods [39]. Plant-based drug-discovery models such as Phyto-MAP further show that systematic screening requires authentic, traceable, and environmentally safe plant material [39]. Therefore, Ayurvedic pharmacological quality control should integrate botanical authentication, phytochemical profiling, toxicological screening, contamination testing, digital traceability, conservation-linked documentation, and sustainable cultivation. Sustainable cultivation is especially important because it can reduce pressure on wild populations while supporting reliable raw material supply, quality consistency, and long-term conservation [19]. Sustainable sourcing and quality-control approaches strengthen medicinal plant authentication, safety, and conservation (Table 3).

**Table 3:** Sustainable sourcing, authentication, and quality-control strategies

Strategy / Quality Measure	Main Purpose	Environmental Health Relevance	Reference
Chemosensory-based authentication	Converts Ayurvedic sensory classification into measurable analytical fingerprints.	Reduces ambiguity, misidentification, and substitution risk.	[16]
Digital Ayurvedic knowledge integration	Connects formulations, medicinal plants, phytochemicals, and diseases.	Supports traceability and transparency of plant–compound-disease relationships.	[18]
Toxicity screening	Identifies preliminary safety risks in medicinal plant extracts.	Helps detect potentially toxic preparations before wider use.	[20]
Agro-ecological cultivation	Promotes biodiversity-sensitive medicinal plant production.	Reduces wild-harvesting pressure and supports ecological resilience.	[32]
Efficacy-oriented quality grading	Grades herbal medicines using efficacy-related quality indicators.	Improves quality-linked procurement and raw material reliability.	[33]
Botanical repositories	Preserves reference specimens for authentication and conservation.	Links biodiversity conservation with medicinal plant identity verification.	[34]

Discussion, policy implications, and future directions

The growth of Ayurvedic pharmacology has created opportunities for drug discovery, integrative healthcare, and herbal therapeutic development but has also increased pressure on medicinal plant supplies [3]. Traditional medicines have gained renewed attention in biomedical research, including infectious disease management and immune support [40]. Based on the evidence discussed in this review, policy recommendations should remain focused on the main risks affecting medicinal plant quality, safety, and sustainability: overharvesting, poor authentication, contamination, adulteration, and inconsistent raw material quality. Increased scientific and commercial interest may raise demand for selected plant species and phytochemicals, making sustainable sourcing essential for Ayurvedic medicine [13]. Without sustainable procurement, this expansion may compromise both ecological stability and herbal product reliability [16]. Policies supporting Ayurvedic medicinal plants should therefore prioritise biodiversity conservation, sustainable cultivation, pharmacological evaluation, toxicology screening, contaminant testing, and environmental health safeguards. Agro-ecological practices provide a relevant model because they link medicinal plant cultivation with biodiversity protection, farmer participation, and production of quality raw materials [32]. Such practices can reduce reliance on wild collection and promote consistency in medicinal plant material [16]. Policy measures should include cultivation support, nursery development, conservation farming, collector training, certification of sustainably sourced plants, routine toxicology screening, heavy metal and pesticide-residue testing, microbial-quality assessment, and measures to detect and minimise adulteration [32].

Quality governance must also emerge as a major policy concern. The use of plant-based drug discovery strategies, such as Phyto-MAP for the AYUSH heritage, highlights the need for systematic identification, phytochemical profiling, and disease-focused screening [39]. In such cases, plant identification, proper procurement, and contamination-free material are crucial [39]. Research related to the use of green-synthesised nanoparticles and medicinal plants in lung carcinoma further highlights the importance of using plant sources in cutting-edge studies [14]. In the future, Ayurvedic pharmacology must consider possible environmental and occupational health hazards associated with medicinal plant procurement processes. Collectors, cultivators, and small-scale distributors may have to work in hazardous conditions and suffer exposure to toxic substances, unstable environments, and economic uncertainties [16]. Policy considerations for sustainable sourcing can thus include adequate compensation, participation, benefit sharing, and recognition of traditional ecological knowledge of medicinal plant resources [26].

The current trend for scientific research in the field of Ayurveda ought to be oriented toward not only pharmacological evaluation but also sustainability-related criteria and methods [19]. Metabolites of plants that may help prevent and treat cardiovascular diseases were studied; thus, it is possible to see how bioactive plant-derived substances are of particular significance [19]. In turn, stilbenes are presented as substances found in plants and going from folklore to pharmaceutics [41]. As seen from the example provided, the significance of traditional plant-based knowledge in pharmacology is apparent; however, further research must focus on sustainability [41]. The ecological footprint should be assessed alongside pharmacological potential [19]. Future research could be dedicated to topics such as digital traceability, ecological risk assessment, botanical collections, species-specific conservation, and quality grading based on therapeutic properties [23]. It has been shown in natural product studies that there are various applications for antioxidants, anti-inflammatories, anticancer drugs, and skincare products [42]. Such applications will be sustainable only if the sources are capable of preserving biodiversity, avoiding contamination, and maintaining raw material purity [40,42]. Thus, Ayurvedic pharmacology needs to develop into a science that is environmentally accountable in terms of conservation, quality, and healthcare and pharmaceutical development [3].

Limitations and future directions

This review has several limitations. As a narrative review, it did not follow a formal systematic review protocol, and no quantitative meta-analysis or structured risk-of-bias assessment was performed. Although a structured search strategy was used, study selection involved narrative judgement; therefore, selection bias is possible. The review may also have missed relevant studies because the literature was selected according to thematic relevance rather than predefined systematic review criteria. In addition, this review is based on existing literature and does not include field-based ecological assessments, toxicological surveillance, or supply-chain audits. Most available studies focus on pharmacological activity, phytochemical composition, or therapeutic relevance, while fewer examine harvesting pressure, habitat vulnerability, contamination exposure, or trade-related sustainability risks. Conservation data for many medicinal plant species also remain inconsistent, limiting the ability to conduct comprehensive ecological risk assessments.

Future research should integrate Ayurvedic pharmacology with conservation biology, environmental toxicology, agroecology, and supply-chain governance. Species-specific studies are needed to assess harvesting pressure, regeneration capacity, habitat sensitivity, cultivation feasibility, and substitution risk. Long-term monitoring should evaluate heavy metals, pesticide residues, microbial contamination, adulteration, and post-harvest quality changes. Traceability systems, botanical repositories, genomic authentication, efficacy-oriented quality grading, and community-level conservation models may strengthen both medicinal plant quality and environmental accountability.

## Conclusions

Sustainable medicinal plant sourcing is essential for the safety, reliability, and environmental accountability of Ayurvedic pharmacology. Growing interest in Ayurveda has increased demand for botanicals used in chronic, inflammatory, metabolic, neurodegenerative, respiratory, infectious, cancer-related, dermatological, and cosmetic applications. This expansion supports innovation in herbal medicine and drug discovery, but the evidence base remains uneven across therapeutic areas. Some claims are supported mainly by traditional use, phytochemical evidence, computational studies, or preclinical mechanisms, while fewer are supported by robust clinical evidence. Therefore, therapeutic relevance should be interpreted according to evidence strength and not assumed from mechanistic activity alone. The reviewed literature indicates that increasing demand may intensify overharvesting, habitat degradation, biodiversity loss, adulteration, contamination, and raw material variability. Environmental sustainability should therefore be treated as one determinant of medicinal plant quality, alongside botanical authentication, phytochemical standardisation, toxicology screening, contamination testing, dose consistency, and post-market quality control.

Cultivation-based sourcing, agro-ecological production, conservation of threatened species, traceable supply chains, and community participation are practical strategies for reducing ecological pressure while improving raw material reliability. Integrating conservation science with Ayurvedic pharmacology may support the production of authentic, safe, and therapeutically consistent herbal medicines, provided that sustainability measures are combined with rigorous quality control and appropriately graded clinical evidence.
